# Facteurs associés à une première grossesse chez les femmes Malgaches ayant accouché dans un hôpital de maternité d'Antananarivo: une étude de cohorte rétrospective

**DOI:** 10.11604/pamj.2018.29.32.13169

**Published:** 2018-01-16

**Authors:** Eddie Rekoronirina, Justin Rahariniaina, Fanjandrainy Rasoaherinomenjanahary

**Affiliations:** 1 Faculté de Médecine, Université d'Antananarivo, Antananarivo 101, Madagascar; 2 Pavillon Sainte Fleur, Hôpital CHU/JRA Ampefiloha, Antananarivo 101, Madagascar; 3 PSI Madagascar Immeuble Fiaro Escalier D 2^ème^ étage Ampefiloha, Antananarivo 101, Madagascar, Population Services International, 1120 19th St NW Suite 600, Washington, DC 20036, USA; 4 Service de Chirurgie viscérale B - CHU-JRA Antananarivo, Madagascar

**Keywords:** Accouchement, césarienne, facteurs de risques, primigestes, Childbirth, cesarean section, risk factors, primigravidae

## Abstract

**Introduction:**

Il y a très peu de littératures africaines et Malgaches concernant les détails sur les facteurs de risques qu'encourent les primigestes en général. Le but de notre étude est de déterminer les facteurs de risques potentiels associés à une première grossesse.

**Méthodes:**

Une étude cohorte rétrospective a été menée auprès des femmes primigestes et multigestes de l'hôpital Pavillon sainte Fleur entre Octobre 2014 et Décembre 2016. Les risques relatifs étaient ajustés après contrôle avec les caractéristiques sociodémographiques.

**Résultats:**

Les primigestes étaient beaucoup plus exposées à un travail prolongé de plus de 12h (RRa = 2,28; IC 95% 1,74-3,00), à une césarienne en urgence (RRa = 1,47; IC 95% 1,35-1,60) et à une épisiotomie (RRa = 2,98; IC 95% 2,61-3,40). Leurs enfants étaient plus susceptibles de présenter des signes de souffrance fœtale avec anomalie du rythme cardiaque fœtale au cours de la phase de travail (RRa = 1,96; IC 95% 1,45-2,65) et un risque accru d'être admis dans une unité de soins intensifs après l'accouchement (RRa = 2,08; IC 95% 1,25-3,45).

**Conclusion:**

Les complications survenues pendant le travail auraient exposé les primigestes à d'autres risques en cascades sur l'issue de leurs accouchements et sur la santé de leurs enfants. La prise en charge des primigestes nécessiterait, de la part des personnels médicaux, une attention particulière sur la durée de la phase de travail.

## Abstract (en)

**Introduction:**

There are very few African and Malagasy literatures on general risk factors among primigravidae. This study aimed to determine the potential risk factors associated with a first pregnancy.

**Methods:**

We conducted a retrospective cohort study among primigravidae and multigravidae women at the Hospital Sainte Fleur Pavilion between October 2014 and December 2016. The relative risks were adjusted after examinations according to sociodemographic features.

**Results:**

Primigravidae were far more likely to experience prolonged labour lasting more than 12h (aRR = 2.28; 95% CI 1.74-3.00), emergency cesarean section (RR = 1.47; 95% CI 1.35-1.60) and episiotomy (aRR = 2.98; 95% CI 2.61-3.40). Children were more likely to show signs of fetal distress associated with fetal rhythm abnormalities during labor (aRR = 1.96; 95% CI 1.45-2.65) and an increased risk for intensive care unit admission after birth (aRR = 2.08; 95% CI 1.25-3.45).

**Conclusion:**

Complications arising during labor may expose primigravidae to cascading risks concerning delivery outcomes and children's health. The management of labour in primigravidae should require close attention from medical staff.

## Introduction

Une première grossesse est une toute nouvelle expérience pour les femmes. En effet, outre l'impact psychologique généré par la peur associée à la grossesse des primigestes [1], les enfants issus de ces primigestes sont également exposés à un certain niveau de risque de décès. L'Enquête Nationale sur le suivi des Objectifs du Millénaire pour le Développement à Madagascar (ENSOMD) conduit en 2012-2013 a révélé que « un enfant appartenant à une catégorie à risque inévitable, c'est-à-dire chez les primigestes âgées de 18 à 34 ans, présente un risque de décéder 1,15 fois plus élevé que celui de la catégorie de référence constituée par les enfants n'appartenant à aucune catégorie de risques considérés » [2]. Nous estimons qu'en amont du risque de décès de l'enfant, plusieurs risques sont liés aux primigestes, aussi bien tout au long de leur grossesse, pendant la phase de travail que durant l'accouchement. La littérature est très pauvre concernant les risques en général qu'encourent les primigestes aussi bien en Afrique qu'à Madagascar. Ainsi, connaitre et comprendre ces facteurs seraient un atout pour mieux gérer le cas de ces primigestes. L'hôpital « Pavillon Sainte Fleur » (PSF), qui est une maternité de référence située au sein de l'hôpital universitaire HJRA d'Antananarivo s'avérait alors un milieu approprié pour réaliser notre étude. Cet hôpital assure le suivi entier d'une grossesse et de l'enfant dont entre autre la consultation prénatale, la préparation et l'assistance à l'accouchement, le suivi et la vaccination de l'enfant dans sa première année. Connu pour ses qualités de service, ce centre reçoit des appuis d'ordre techniques et financiers par l'association « Ordre de malte » de France en partenariat avec le gouvernement Malgache. L'objectif de notre étude est de déterminer les facteurs associés à une première grossesse en vue d'aider les personnels médicaux à mieux se préparer face aux risques potentiels ainsi que d'informer et préparer à l'avance les parturientes.

## Méthodes

**Type d'étude**: il s'agit d'une étude de cohorte rétrospective utilisant les données collectées systématiquement auprès des femmes enceintes pour la première fois, dont le suivi de grossesse et l'accouchement se sont déroulés dans l'hôpital PSF.

### Population d'étude

Toutes les femmes enceintes ayant donné naissance au sein de l'établissement PSF d'Octobre 2014 à Décembre 2016 ont été incluses dans l'étude. Elles ont été réparties en groupe de primigestes et en groupes de multigestes puis ont été suivi rétrospectivement tout au long de leur grossesse jusqu'à leur accouchement. Les bébés nés de cet accouchement ont été également suivis afin de déterminer les éventuels risques pour les bébés. Les femmes enceintes qui ont commencé leur consultation prénatale (CPN) auprès de l'établissement mais qui ont été perdues de vue et dont l'issue de l'accouchement est inconnue, ont été exclues de l'étude.

### Collecte des données

Depuis Octobre 2014, l'établissement PSF collecte systématiquement les données des femmes enceintes utilisant un questionnaire structuré qui a été préalablement pre-testé et amélioré avant son utilisation effective. Les données ont été saisies périodiquement dans un formulaire électronique développé avec Epidata 3.1 (The EpiData Association, Odense Denmark).

### Analyse des données

La gestion et l'analyse des données ont été effectuées avec Stata 13.1 (StataCorp©, College Station, TX). Une analyse comparative des caractéristiques sociodémographiques des primigestes et des multigestes a été effectuée en utilisant le test chi2 exact de Fischer et l'odds ratio a été rapporté. Les facteurs associés à la mère tout au long de sa grossesse jusqu'à son accouchement ont été analysés en comparant les primigestes et les multigestes tandis que les facteurs associés à l'enfant ont été analysés en comparant chaque bébé. Pour chaque facteur étudié, les valeurs manquantes ont été exclues de l'analyse. L'analyse des facteurs associés a été contrôlée avec l'âge, le statut matrimonial, le niveau d'étude et la profession considérés comme des facteurs de confusion. L'analyse des facteurs de confusion et de modificateur d'effet était vérifiée avec le test d'homogénéité de Mantel-Haenszel. Le test Chi2 exact de Fischer a été utilisé dans l'ensemble des analyses avec un seuil de significativité à 0,05. Les valeurs brutes et les valeurs ajustées des risques relatifs (RR) pour chaque facteur étudié ont été rapportées.

### Ethiques et protection des sujets humains

L'étude respecte la déclaration d'Helsinki. Des mesures ont été mises en place pour maintenir la confidentialité et l'anonymat des femmes incluses dans l'étude.

### Limite de l'étude

Il s'agit d'une étude effectuée en milieu hospitalier et en milieu urbain. Elle pourrait ne pas être représentative de toutes les primigestes dans tout le territoire malgache, entre autre celles qui ne venaient pas faire leur CPN dans un établissement sanitaire et de celles vivant dans le milieu rural. Les valeurs manquantes de certains facteurs étudiés pourraient avoir entrainé un biais de catégorisation entre les primigestes et les multigestes.

## Résultats

Sur 5924 femmes enceintes incluses dans l'étude dont 1813 étaient des primigestes représentant une proportion de 30,60%. L'âge moyen des primigestes était de 25,41 ans et celui des multigestes était de 30,35 ans. Effectivement, les primigestes avaient un profil de femme de moins de 25 ans (43,91%; OR = 5,36; p < 0,001); célibataire (13,71%; OR = 1,95; p < 0,001); avec un niveau d'éducation universitaire (60,53%; OR = 1,43; p < 0,001) et ayant un emploi avec un revenu régulier (69,26%; OR = 1,16; p = 0,014) (Tableau 1).

**Tableau 1:** Profil sociodémographique des primigestes

	Non primigesteEffectif (%)	PrimigesteEffectif (%)	Odds ratio(IC 95%)	p
Age	N=4111	N=1813		
Moins de 25 ans	523 (12,72)	796 (43,91)	**5,36** **(4,71-6,11)**	**p<0001**
Situation matrimoniale	N=3771	N=1677		
Célibataire	283 (7,50)	230 (13,71)	**1,95** **(1,62-2,35)**	**p<0001**
Niveau d’éducation	N=3456	N=1586		
Niveau universitaire	1787 (51,71)	960 (60,53)	**1,43** **(1,26-1,61)**	**p<0001**
Profession	N=3874	N=1737		
Ayant un emploi avec un revenu régulier	2556 (65,93)	1204 (69,26)	**1,16** **(1,03-1,31)**	**p=0014**

Les estimations en gras indique un test d’association significative avec p<0,05

### Facteurs associés à la mère

Bien qu'une première CPN tardive après 22 semaines d'aménorrhées (SA) était apparemment un des facteurs de risques chez les primigestes, ce facteur s'est montré non significatif après contrôle avec les caractéristiques socio-démographiques. Par contre, une longue durée de travail après rupture des poches des eaux était très particulière chez les primigestes où le niveau de risque est croissant en fonction de l'étendue de la durée. En effet, le risque pour les primigestes d'accoucher plus de 4h après la rupture des poches des eaux était de 1,62 (IC 95% 1.40-1.89). Ce risque augmente à 2,05 (IC 95% 1,66-2,55) pour un accouchement après 8h et même jusqu'à 2,28 (IC 95% 1,74-3,00) pour un accouchement après 12h. Ce constat est en concordance avec les risques pour les primigestes de bénéficier d'un déclenchement de travail (RRa = 1,52; IC 95% 1,30-1,79), les risques de dystocie nécessitant un recours au matériel d'extraction comme le vacuum(RRa = 2,20; IC 95% 1,79-2,71) ainsi que le risque de recourir à une césarienne d'urgence (RRa = 1,47; IC 95% 1,35-1,60) ou une césarienne après échec de la tentative d'accouchement par voie basse (RRa = 2,61; IC 95% 2,20-3,10). Durant la phase de travail, les primigestes étaient beaucoup plus exposées aux signes en faveur d'une souffrance fœtale dont un liquide amniotique teinté, méconial ou fétide (RRa = 1,29; IC 95% 1.17-1.43) et un rythme cardiaque fœtal anormal détecté lors du monitorage (RRa = 1,96; IC 95% 1,45-2,65). Finalement, le risque le plus élevé associé aux primigestes était celui de subir une épisiotomie quand elles accouchaient par voie basse (RRa = 2,98; IC 95% 2,61-3,40) (Tableau 2). A côté de ces facteurs de risques suscités, certains avantages étaient associés aux femmes qui étaient enceintes pour la première fois de leur vie. En effet, les primigestes étaient moins susceptibles d'être en surpoids (RRa = 0.65; IC 95% 0.54- 0.78), ou d'être obèse (RRa=0,50; IC 95% 0,30-0,83) ou diabétique(RRa = 0,45; IC 95% 0,24-0,86). Elles étaient également moins exposées à une césarienne en générale (RRa = 0,90; IC 95% 0,82-0,99) et étaient plus susceptibles d'accoucher après 38 SA (RRa = 1,19; IC 95% 1,11-1,29) sans toutefois être a risque d'avoir un enfant post mature. Enfin, elles étaient beaucoup plus disposées à assister à plus de 4 CPN comparés aux multigestes (RRa=1,04; IC 95% 1,00-1,08) (Tableau 3).

**Tableau 2:** Facteurs de risques associés aux mères primigestes

	Non primigesteEffectif (%)	PrimigesteEffectif (%)	RR brute (IC 95%)	RR ajusté (IC 95%)
Première CPN	N=3650	N=1637		
Première CPN>22SA	1283 (35,15)	689 (42,09)	**1,19 (1,11-1,28)**	1,01 (0,93-1,10)
Durée de travail après rupture des poches des eaux jusqu’à l’accouchement	N = 2873	N=1180		
Plus de 4h	440 (15,32)	310 (26,27)	**1,71 (1,50-1,95)**	**1,62 (1,40-1,89)**
Plus de 8h	195 (6,79)	169 (14,32)	**2,11 (1,74-2,55)**	**2,05 (1,66-2,55)**
Plus de 12h	123 (4,28)	122 (10,34)	**2,41 (1,90-3,05)**	**2,28 (1,74-3,00)**
Fièvre pendant le travail	N = 4111	N=1813		
Fièvre pendant le travail	51 (1,24)	45 (2,48)	**2,00 (1,35-2,95)**	**1,95 (1,27-2,97)**
Liquide amniotique	N = 4094	N=1807		
Teinté ou Méconial ou Fétide	1082 (26,43)	579 (32,04)	**1,21 (1,11-1,32)**	**1,29 (1,17-1,43)**
Rythme cardiaque fœtale au monitoring pendant le travail	N = 4000	N=1764		
Rythme cardiaque anormal	121 (3,02)	101 (5,73)	**1,89 (1,46-2,44)**	**1,96 (1,45-2,65)**
Déclenchement pendant le travail	N=3361	N=1691		
Ayant subi un déclenchement	394 (11,72)	308 (18,21)	**1,78 (1,55-2,04)**	**1,52 (1,30-1,79)**
Utilisation de matériel d’extraction *	N = 2602	N=1269		
Ayant subi un vacuum ou autres	217 (8,34)	244 (19,23)	**2,30 (1,95-2,72)**	**2,20 (1,79-2,71)**
Césarienne **	N = 1450	N=522		
Césarienne en urgence	732 (50,48)	390 (74,71)	**1.47 (1.36-1.60)**	**1.47 (1.35-1.60)**
Césarienne après échec de tentative par voie vaginale	224 (15,45)	219 (41,95)	**2,71 (2,32- 3,17)**	**2,61 (2,20-3,10)**
Episiotomie *	N = 2581	N=1260		
Ayant subi une épisiotomie	341 (13,21)	612 (48,57)	**3,67 (3,30-4,09)**	**2,98 (2,61-3,40)**
Hémorragie *	N = 2587	N=1261		
Hémorragie à l’accouchement	58 (2,24)	30 (2,38)	1,06 (0,68-1,64)	1,31 (0,75-2,30)
Révision utérine *	N = 2595	N=1264		
Révision utérine après accouchement	1802 (69,44)	950 (75,16)	**1,19 (1,12-1,25)**	**1,13 (1,06-1,21)**

RR ajustés résultaient d’un contrôle avec l’âge, la situation matrimoniale, le niveau d’étude et la profession ; les estimations en gras indique un test d’association significative avec p<0,05

* Parmi celles qui ont accouché par voie basse

** Parmi toutes les césariennes mais avec exclusion de celles par convenance. L’échec de tentative par voie basse incluait un échec du déclenchement, dystocie mécanique, stagnation de la dilatation du col utérin

**Tableau 3:** Facteurs à l’avantage des mères primigestes

	Non primigesteEffectif (%)	PrimigesteEffectif (%)	RR brute (IC 95%)	RR ajusté (IC 95%)
Nombre de CPN	N = 3970	N = 1757		
Nombre de CPN >=4	3066 (77,23)	1402 (79,80)	**1,03 (1,00-1,06)**	**1,04 (1,00-1,08)**
Poids habituel ₹	N = 2367	N = 1170		
Surpoids	581 (24,55)	150 (12,82)	**0,52 (0,44-0,61)**	**0,65 (0,54- 0,78)**
Obèse	102 (4,31)	22 (1,88)	**0,43 (0,28-0,67)**	**0,50 (0,30-0,83)**
Hospitalisation avant l’accouchement	N = 4058	N = 1794		
A n’importe quel moment de la grossesse	124 (3,06)	50 (2,79)	0,91 (0,66-1,26)	1,16 (0,79-1,70)
Menace d’avortement ou d’accouchement prématuré	N = 4057	N=1794		
Existence de la menace	57 (1,40)	29 (1,62)	1,15 (0,73-1,79)	1,44 (0,85-2,43)
Tension artérielle	N = 4065	N = 1796		
HTA	161 (3,96)	52 (2,90)	**0,73 (0,53-0,99)**	0,87 (0,59-1,29)
Glycémie	N = 4045	N = 1784		
Diabète	84 (2,08)	14 (0,78)	**0,37 (0,22-0,64)**	**0,45 (0,24-0,86)**
Pré-éclampsie	N = 4065	N = 1796		
Pré-éclampsie	82 (2,02)	26 (1,45)	0,71 (0,46-1,10)	0,88 (0,51-1,52)
Terme	N = 4057	N = 1794		
Prématurité	546 (13,46)	217 (12,10)	0,89 (0,77-1,04)	0,94 (0,79-1,13)
Terme après 38 SA	1605 (39,56)	838 (46,71)	**1,18 (1,10-1,25)**	**1,19 (1,11-1,29)**
Terme après 39 SA	722 (17,80)	376 (20,96)	**1,17 (1,05-1,31)**	**1,14 (1,00-1,30)**
Terme après 40 SA	142 (3,50)	72 (4,01)	1,14 (0,86-1,51)	1,29 (0,91-1,83)
Post-maturité	14 (0,35)	5 (0,28)	0,80 (0,29-2,23)	0,40 (0,07-2,16)
Mode d’accouchement $	N = 4054	N = 1792		
Césarienne en général	1452 (35,82)	523 (29,19)	**0,81 (0,75-0,88)**	**0,90 (0,82-0,99)**

RR ajustés résultaient d’un contrôle avec l’âge, la situation matrimoniale, le niveau d’étude et la profession ; les estimations en gras indique un test d’association significative avec p<0,05

$ Les césariennes par convenance ont été exclues du dénominateur

₹ Selon l’OMS, le surpoids est défini par IMC>=25 et l’obésité par IMC>=30

### Facteurs associés à l'enfant

Avec les 200 jumeaux et les 9 triplets inclus, 6030 enfants ont été étudiés au total dont 1836 (30,45%) d'entre eux étaient nés de mères primigestes. Bien que les enfants issus des mères primigestes étaient apparemment susceptibles d'avoir un score d'Apgar inférieur à 7 à une minute de la naissance, cette vivacité, ne différaient pas significativement de ceux des mères multigestes après contrôle de ce facteur avec les caractéristiques socio-démographiques. Par contre, les enfants des primigestes s'étaient montrés beaucoup plus nécessiteux d'une admission dans une unité de soins intensifs comparés au groupe de contrôle (RRa=2,08 ; IC 95% 1,25-3,45). Ces enfants issus d'une première naissance avaient également une forte chance de peser moins de 3kg à la naissance (RRa = 1,12; IC 95% 1,05-1,20) même s'ils n'étaient pas à risque d'être de petit poids (inférieur à 2500g). Le risque de mortalité infantile n'était pas significativement diffèrent entre les deux groupes (Tableau 4).

**Tableau 4:** Facteurs associés aux enfants des primigestes

	Non primigesteEffectif (%)	PrimigesteEffectif (%)	RR brute (IC 95%)	RR ajusté(IC 95%)
Vivacité a la naissance^¥^	N=4135	N=1819		
Apgar < 7 à 1 mn	67 (1,62)	45 (2,47)	**1,52 (1,05-2,21)**	1,55 (0,99-2,44)
Admission dans une unité de soins intensif (USI) ^¥^	N=4137	N=1820		
Enfant admis en USI	56 (1,35)	42 (2,31)	**1,70 (1,15-2,52)**	**2,08 (1,25-3,45)**
Allaitement	N=4156	N=1817		
Allaitement effectif dans les 24h après la naissance	4031 (96,99)	1765 (97,14)	1,00 (0,99-1,01)	0,99 (0,98-1,00)
Poids à la naissance	N=4194	N=1836		
Petits poids <2500g	526 (12,54)	261 (14,22)	1,13 (0,98-1,30)	1,16 (0,98-1,37)
Poids <3000g	1881 (44,85)	943 (51,36)	**1,14 (1,08-1,21)**	**1,12 (1,05-1,20)**
Périmètre crânien	N=4194	N=1836		
Microcéphalie ^β^	216 (5,15)	115 (6,26)	1,21 (0,97-1,51)	1,25 (0,97-1,61)
Mortalité infantile	N=4194	N=1836		
Mortalité périnatale	111 (2,65)	37 (2,02)	0,76 (0,52-1,09)	0,95 (0,62-1,47)
Mort in utero	57 (1,36)	16 (0,87)	0,64 (0,37-1,10)	0,90 (0,48-1,68)
Mortalité néonatale	54 (1,29)	21 (1,14)	0,88 (0,53-1,46)	1,00 (0,54-1,85)

RR ajustés résultaient d’un contrôle avec l’âge, la situation matrimoniale, le niveau d’étude et la profession; les estimations en gras indique un test d’association significative avec p<0,05

¥ Les enfants morts in utero ont été exclus du dénominateur

β La microcéphalie a été définit selon les critères de l’OMS dont un périmètre crânien à la naissance inférieur à 30,7 cm chez les garçons et inférieur à 30,3 cm chez les filles

## Discussion

Se référant à d'autres études, la proportion des primigestes et leur profil de jeunes célibataires de moins de 25 ans étaient attendus. Par contre, nous estimons que le profil des primigestes comme étant de niveau universitaire et ayant un revenu régulier reflète tout simplement l'attente des primigestes vis-à-vis de la prise en charge de leur première grossesse à travers l'hôpital sachant que l'hôpital PSF est payant [3-5].

### Facteurs associés à la mère

Notre étude a montré que le travail prolongé après rupture des membranes était habituel chez les primigestes malgré la prédominance significative du déclenchement du travail chez elles comparées aux multigestes. Tout en confirmant cette association avec les primigestes, une étude coréenne concluait que l'aspect en entonnoir de col de l'utérus pourrait être un facteur prédictif de la réussite du déclenchement du travail chez les primigestes [6]. Par ailleurs, ce travail prolongé de plus de 12h chez les primigestes a été également rapporté dans l'étude de Nazia Hashim et al. qui ajoutait que cette différence entre primigestes et multigestes était significative pour toutes les étapes de la phase active du travail [7]. Farhana Shaikh et al associaient de leur côté, le travail prolongé de plus de 12h à la présentation céphalique haute au début du travail des primigestes [8] tandis que d'autres études mentionnait une dilatation lente du col utérin pendant le travail [4,9]. Certains auteurs quant à eux, l'associaient à des facteurs comme la dystocie [10]. Même si ces associations directes n'étaient pas retrouvées dans notre étude, elles pourraient être reflétées, par le recours au vacuum qui a été également citée par certains auteurs comme significativement plus utilisés chez les primigestes [7,11] ou également reflétée par le recours à une césarienne après un échec de la tentative de l'accouchement par voie vaginale, qui a été significativement élevé chez les primigestes de notre étude. Il est important de préciser le fait paradoxal entre la césarienne d'indication médicale en général (excluant celle par convenance) et la césarienne en urgence dans notre étude. En effet, la première a été significativement moins effectuée auprès des primigestes de notre étude bien que certaines études affirmaient le contraire [11] tandis que la deuxième était étroitement liés aux primigestes comme étant un de leur principaux risques, et ceci en accord avec plusieurs études [7,12]. Cette disparité entre la césarienne programmée et celle réalisée en urgence (secondaire ou non à un échec de tentative par voie vaginale) pourrait s'expliquer par la chance des multigestes d'avoir beaucoup plus de césarienne programmée et planifiée selon le risque détecté pendant la grossesse bien longtemps avant le travail; alors que la césarienne des primigestes étaient décidées après l'adaptation des actions à entreprendre selon l'évolution de la phase de travail et non au recours systématique à la césarienne. En effet, Zarko Alfirevic et al. avaient démontré que cette différence du taux de césarienne dans les établissements serait fonction de la présence et du suivi d'un guide de recommandation sur le partogramme ou sur le monitorage du travail dans l'établissement [13]. Cette assertion était appuyée par Pushpa Chetandas et al. pour une décision de césarienne basée sur la cardiotocographie [14]. Concernant la souffrance fœtale révélée chez les primigestes de notre étude, nous pensons que ce sont la dystocie ou le prolongement du temps de travail après rupture des membranes qui seraient à leur origine plutôt que les autres maladies gestationnelles. Ainsi, la fièvre maternelle pendant le travail, l'anomalie de la cardiotocographie au monitorage et le liquide méconial, qui étaient retrouvés également dans d'autres études, seraient la conséquence des complications survenant pendant le travail [7]. Cette supposition est d'autant plus cohérente quand notre étude s'alliait avec les autres études en mettant en exergue que les primigestes ne différaient pas des multigestes concernant les risques pendant la grossesse telles que l'hypertension artérielle, la preéclampsie, la menace d'accouchement prématuré ou la nécessité d'une hospitalisation. Les primigestes étaient même significativement moins exposées au diabète et à certains facteurs de risque de maladie cardio-vasculaire ou de preéclampsie comme l'obésité qui étaient d'ailleurs très rares chez les primigestes [15-17]. Plusieurs études adhéraient au constat sur le risque élevé pour les primigestes de subir beaucoup plus d'épisiotomie comparées aux multigestes [18]. Certaines études avançaient que cette association serait plutôt due à l'existence d'un guide sur la gestion d'une épisiotomie chez les primigestes pour un établissement donné [13]. Cette épisiotomie de routine effectuée chez les primigestes était effectivement soutenue par certains personnels médicaux comme étant une pratique prudente qui préviendraient les complications et faciliteraient l'accouchement [19]. C'est probablement toujours dans cette même optique de prudence que la révision utérine était beaucoup plus pratiquée chez les primigestes. A l'opposé de tous ces risques suscités, il est encourageant de constater dans notre étude que les primigestes étaient les plus assidues à venir effectuer au moins 4 CPN comme il est recommandé par l'Organisation Mondiale de la Santé (OMS), sachant que la chance pour les parturientes d'accoucher dans un établissement sanitaire est fortement liée à leur volonté d'effectuer le nombre minimal requis pour les CPN [20].

### Facteurs associés à l'enfant

Probablement en continuité avec les signes de souffrances fœtales apparues durant la phase de travail, notre étude rejoignait d'autres études qui rapportaient que les enfants des primigestes étaient beaucoup plus à risque d'être admis dans une unité de soins intensifs que ceux des multigestes bien que le score d'Apgar à la naissance des enfants des deux groupes ne présentait aucune différence significative. Toutefois, malgré cette nécessité de soins intensifs, les nouveau-nés des primigestes ne présentaient pas plus de risque de mortalité que ceux des multigestes et il en était de même pour le risque de mortalité périnatale [7,11]. Même si les enfants des primigestes de notre étude étaient plus susceptibles de peser moins de 3kg à la naissance, notre résultat était en accord avec les autres études qui confirmaient l'absence de risque significatif de peser moins de 2,5kg à la naissance de ces enfants des primigestes [11]. Ce risque affecterait plutôt les enfants des primigestes adolescents [21]. Partant des résultats sur l'absence de risque de prématurité et de post-maturité pour les enfants des primigestes de notre étude, l'absence de risque de petit poids à la naissance de ces enfants était également attendue sachant que ces deux facteurs sont étroitement liés. En effet, les études confirmaient également l'absence de différence significative entre les accouchements prématurés des primigestes comparés aux multigestes [11] et attribuaient plutôt ce risque de prématurité aux primigestes adolescents comparées à leurs ainées primigestes [21].

## Conclusion

Nombreux sont les facteurs de risques auxquels étaient exposés les primigestes quel que soit leur âge, leur situation matrimoniale, leur niveau d'étude ou leur profession mais les risques majeurs étaient constitués par les complications intra-partum autour desquelles seraient liées les autres facteurs de risques. En effet, les problèmes survenus au cours du travail tels que la fièvre, la dystocie, le travail prolongé et l'échec de l'utilisation de vacuum auraient exposé les primigestes au risque de subir beaucoup plus de césarienne ou d'épisiotomie en cas d'accouchement par voie vaginale. Ces activités étaient considérées par les praticiens comme une attitude de prudence pour limiter les complications chez les primigestes. Ces complications pendant la phase de travail auraient également des impacts sur les fœtus et les nouveau-nés des primigestes avec la révélation des risques d'anomalies cardiotocographiques lors du monitorage du travail ou de liquide amniotique méconial. Cette souffrance fœtale aurait également exposé ces enfants nés des mères primigestes à la nécessité de plus de surveillance se traduisant par leur admission dans une unité de soins intensifs après leur naissance. Au vu de tous ces risques, il est impératif pour les personnels médicaux d'augmenter leur attention lors de la phase de travail des primigestes et de diminuer autant que possible la durée de travail en ayant recours à une césarienne le plus tôt possible en cas d'échec des tentatives d'extraction fœtale par d'autres moyens instrumentaux.

### Etat des connaissances actuelle sur le sujet

- Une étude auprès des ménages malgaches pour le suivi des objectifs du millénaire pour le développement a montré un certain niveau de risque de décès pour les enfants des primigestes;
- L'impact psychologique généré par la peur pour le bon déroulement de la première grossesse chez les primigestes;
- Les différents risques comparatifs entre primigestes adolescents et primigestes adultes.

### Contribution de notre étude à la connaissance

- L'incidence des différents facteurs associés à la première grossesse des femmes Malgaches;
- Les facteurs de risques à toutes les étapes de la grossesse et à l'accouchement des primigestes Malgaches quels que soient leurs caractéristiques socio-démographiques;
- Les facteurs de risques qu'encourent les enfants nés d'une première grossesse des mères Malgaches.

## Conflits d'intérêts

Les auteurs ne déclarent aucun conflit d'intérêts.

## Remerciements

Les auteurs tiennent à remercier toute l'équipe de l'hôpital PSF qui a contribué de près ou de loin à la réalisation de l'étude. Remerciement spécial à ceux qui avaient assuré successivement la responsabilité de la coordination de la collecte des données ainsi que de la supervision de la saisie des données.
